# Reflectance Spectroscopy as a Novel Tool for Thickness Measurements of Paint Layers

**DOI:** 10.3390/molecules28124683

**Published:** 2023-06-09

**Authors:** Alice Dal Fovo, Marina Martínez-Weinbaum, Mohamed Oujja, Marta Castillejo, Raffaella Fontana

**Affiliations:** 1 Consiglio Nazionale delle Ricerche-Istituto Nazionale di Ottica (CNR-INO), Largo E. Fermi 6, 50125 Florence, Italy; raffaella.fontana@ino.cnr.it; 2 Instituto de Química Física Rocasolano, Spanish National Research Council (CSIC), C/Serrano 119, 28006 Madrid, Spain; mgmartinez@iqfr.csic.es (M.M.-W.); m.oujja@csic.es (M.O.); marta.castillejo@iqfr.csic.es (M.C.)

**Keywords:** paintings, reflectance spectroscopy, OCT, LIBS, Raman spectroscopy, thickness measurements

## Abstract

A major challenge in heritage science is the non-invasive cross-sectional analysis of paintings. When low-energy probes are used, the presence of opaque media can significantly hinder the penetration of incident radiation, as well as the collection of the backscattered signal. Currently, no technique is capable of uniquely and noninvasively measuring the micrometric thickness of heterogeneous materials, such as pictorial layers, for any painting material. The aim of this work was to explore the possibility of extracting stratigraphic information from reflectance spectra obtained by diffuse reflectance spectroscopy (DRS). We tested the proposed approach on single layers of ten pure acrylic paints. The chemical composition of each paint was first characterised by micro-Raman and laser-induced breakdown spectroscopies. The spectral behaviour was analysed by both Fibre Optics Reflectance Spectroscopy (FORS) and Vis-NIR multispectral reflectance imaging. We showed that there is a clear correlation between the spectral response of acrylic paint layers and their micrometric thickness, which was previously measured by Optical Coherence Tomography (OCT). Based on significant spectral features, exponential functions of reflectance vs. thickness were obtained for each paint, which can be used as calibration curves for thickness measurements. To the best of our knowledge, similar approaches for cross-sectional measurements of paint layers have never been tested.

## 1. Introduction

In recent decades, a wide variety of scientific techniques have been tested and optimized for the study of cultural heritage (CH) objects. The need to preserve the material integrity of works of art has directed the research toward defining non-invasive analytical approaches based on the combined application of methodologies that do not involve sampling or risk of damage to the object. A major challenge in heritage science is the non-invasive cross-sectional analysis of the pictorial stratigraphy in paintings. Thickness measurement of the micrometric layers is essential, for instance, to monitor the removal of surface materials during the cleaning operation [1] or to assess the compactness and adhesion between layers. In a non-invasive approach, when low-energy probes are used, the presence of opaque media can significantly hinder the penetration of the incident radiation, as well as the signal collection from within the examined materials.

One of the most widely used techniques for non-invasive stratigraphic measurements on paintings is Optical Coherence Tomography (OCT) [2,3]. Primarily applied in the field of ophthalmology, OCT is an interferometric method based on a Michelson interferometer, yielding 2- or 3-dimensional tomographic imaging that allows the visualization of the internal structure of pictorial layers with an axial resolution ranging from 1 to 10 μm (in air). The incident radiation is backscattered by the material and the optical interference is observed whenever the signal superposes with the reference beam, within the coherence length of the light source. The measurement is based on the detection of signals generated at the interfaces between different media—i.e., when the incident radiation experiences a refractive index (n) mismatch. OCT has proven particularly effective in probing materials that are semi-transparent in the near-infrared (NIR) spectral range. This includes most varnishes applied by artists on the painting surface with a protective and/or aesthetic function [4]. By combining an OCT setup with confocal microscope optics, which enables the beam focussing inside the material rather than on the outer surface, even highly reflecting coatings can be measured [5]. Pictorial layers, however, are often composed of pigments with dispersion and/or absorption properties that do not allow their thickness to be assessed by OCT. Moreover, the n-mismatch causes a delay in the optical path of the reference beam and, therefore, the optically measured distances must be corrected to geometrical distances by dividing them by the refractive index of the material. In the NIR, the refractive index of semi-transparent materials used in paintings is conventionally given as 1.5. However, pictorial layers (pigment dispersed in the binder) are often highly heterogeneous and exhibit variable optical properties that result in different n values. Therefore, if n is not known, the correct thickness of the painting layers is not achievable.

In recent decades, alternative methods to OCT have been proposed for the non-invasive in-depth analysis of paintings. Among others, Terahertz imaging [6,7] has proven effective in yielding 3D data sets of interfaces and projections of paintings in the presence of absorbing species. However, the low axial resolution achievable with THz radiation makes this method unfit for micrometric measurements of pictorial layers.

The use of Nuclear Magnetic Resonance (NMR) to obtain stratigraphic information on easel and wall paintings is also well-documented in the literature [8,9]. NMR profiling was tested to investigate both signal intensity and transverse relaxation time distribution, showing that the dependence of signal intensity on relaxation times makes the interpretation of the stratigraphic information difficult [10]. The NMR-sensitive volume averages the effect of irregularities in the layers and the signal from adjacent layers. In addition, the application of this method is hampered by the lack of application-specific operating software, while the low mass sensitivity resulting from low NMR frequencies results in long measurement times. 

More recently, Nonlinear Optical (NLO) techniques [11] have been successfully used for cross-sectional analysis of a wide variety of artistic materials, including paint and varnish samples. The combined application of different NLO modalities allows for the acquisition of compositional and structural information based on the detection of fluorophores (by Multi-Photon Excitation Fluorescence, MPEF), crystalline or highly organized structures without inversion symmetry (by Second Harmonic Generation, SHG), or local differences in refractive index, i.e., interfaces (by Third Harmonic Generation, THG). While for MPEF and SHG, the main limitation in the stratigraphic analysis of paintings is the presence of highly diffusing and/or absorbing media (pigments) [12], the applicability of THG is confined to layers of transparent material, forward detection being the only possible configuration [13].

A cutting-edge methodology recently proposed for cross-sectional analysis in paintings is photoacoustics, which in a sense, can be considered complementary to OCT, as it takes advantage of the presence of non-transparent materials [14,15]. Acoustic waves are generated by the absorption of the radiation emitted by an intensity-modulated pulsed laser. The exponential attenuation of acoustic waves in the frequency domain, which depends on the absorption coefficient of the medium and the propagation path, can be exploited to measure the thickness of the examined material. Although early applications reveal the technique’s potential [16], to date, its use is limited to specific cases only.

Given the above, it can be stated that, at present, there is no technique that can uniquely and non-invasively measure the thickness of heterogeneous and optically opaque materials such as pictorial layers.

In this work, we explored the feasibility of achieving stratigraphic information of painting layers from their reflectance spectra measured by Diffuse Reflectance Spectroscopy (DRS) [17]. In heritage science, DRS is typically applied for the analysis of paintings in multi- and hyper-spectral imaging modes or using fibre optics for point-wise measurements. The main objective is typically the identification and mapping of pigments and binders based on the absorption properties of electronic and vibrational transitions of molecules [18]. In the imaging mode, the use of the NIR spectral range allows for the visualization of hidden details underneath the painted surface related to the artistic working process, such as underdrawings and underpaintings. Diffuse reflectance is defined as the ratio of the irradiance of light reflected back to the detector to the irradiance on the surface of the object, as a function of wavelength. The measured light backscattered from the object includes contributions from the air/surface interface and varies with illumination and collection geometry.

The spectral reflectance behaviour of pigments and pictorial layers has been extensively studied [19,20,21]. It has been shown that the reflectance signal measured from pigment mixtures in paintings is the result of the nonlinear combination of the reflectance of the individual pigments. To cope with the complexity of spectral data interpretation, as well as to reduce the high dimensionality of DRS imaging datasets, new approaches based on artificial intelligence (AI), e.g., deep neural networks (DNN), have been recently explored [22].

In this preliminary study, we tested the proposed approach by examining single layers of pure paint, thus avoiding the use of optical models to predict the reflectance of pigments in mixtures [23] and not taking into account the influence of the surface roughness on the spectra [24]. A mock-up was created for this specific purpose: ten acrylic paints were laid with increasing thicknesses, ranging from 50 to 350 μm, on both white and black backgrounds. The chemical composition of each paint was first characterised by micro-Raman [25] and laser-induced breakdown spectroscopy (LIBS) [26]. The thickness of each layer was then measured by OCT, taking as a reference the portion of the visible substrate at the edge of the paint layer. The spectral behaviour was analysed by both Fibre Optics Reflectance Spectroscopy (FORS) and Vis-NIR multi-spectral reflectance imaging. Finally, for each acrylic paint, meaningful spectral features were identified to assess the dependence of the reflectance on the layer thickness, thus obtaining non-linear fitting curves.

To the best of our knowledge, no similar approaches have been explored before for cross-sectional measurements of paint layers.

## 2. Results

### 2.1. Chemical Characterization of the Acrylic Paints with LIBS and Micro-Raman Spectroscopy

The chemical composition of the ten acrylic paints was assessed by LIBS and micro-Raman spectroscopies (Table 1), using the information reported in the literature [27,28,29,30,31,32] and NIST [33] and IRUG [34] databases. Further information on the chemical composition of the phthalocyanine paints (PBC, PBL and PGL) can be found in our previous work [35].

**Figure 1:**
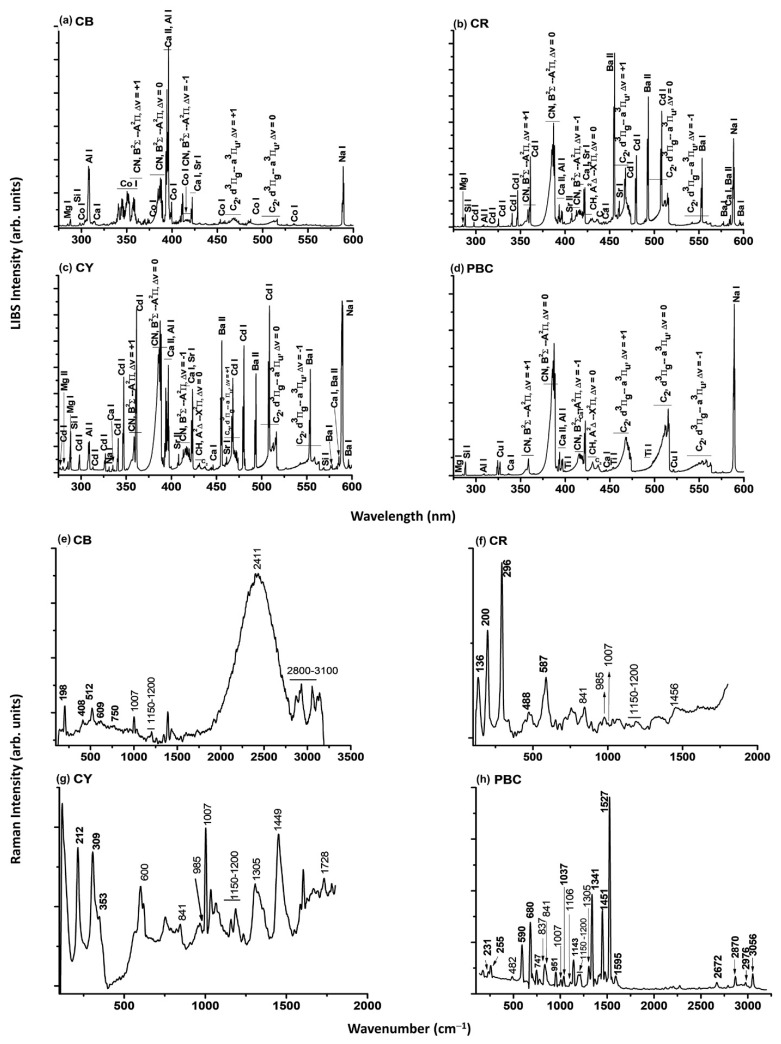
LIBS (top) and micro-Raman (bottom) spectra of cobalt blue (**a**,**e**), cadmium red (**b**,**f**), cadmium yellow (**c**,**g**) and primary blue cyan (**d**,**h**) acrylic paintings, respectively. The micro-Raman spectra are baseline subtracted.

Figure 1 shows the LIBS (top) and micro-Raman (bottom) spectra of cobalt blue (CB), cadmium red (CR), cadmium yellow (CY) and primary blue cyan (PBC) acrylic paints. The spectra measured on the other analysed paints are displayed in Figure S1 in the Supplementary Material. For all paints, the chemical composition (Table 1) agrees with what was declared by the manufacturer (Table 2), except for the absence of titanium dioxide in permanent green light (PGL) and the presence of additional components in most of the analysed paints, ascribed to the binder and fillers. Specifically, atomic emissions of Mg, Si, Ca, Al, Sr, Ba and Na detected by LIBS are ascribed to fillers such as kaolin (Al_2_Si_2_O_5_(OH)_4_), gypsum (CaSO_4_·½H_2_O), carbonates (CaCO_3_, MgCO_3_), glass powder and barite (BaSO_4_) [31]. The molecular bands of CN (Violet band), CH and C_2_ (Swan bands) are due to the acrylic binder, as well as to the organic pigments. The LIBS results obtained in these findings agree with the ones by micro-Raman spectroscopy. Bands from calcium sulfate (1007 cm^−1^), calcium carbonate (1085 cm^−1^) and barium sulfate (985 cm^−1^) are observed in each acrylic paint and attributed to the fillers. Additional bands at 482, 600, 620, 837, 841, 1106, 1150–1200, 1240, 1305, 1449, 1452, 1728, 2411 and 2800–3100 cm^−1^ are attributed to the constituents of the polymeric binder [27,28,29,30,31,32].

LIBS results reported in Table 1 show the presence of the main paintings markers such as Co for cobalt blue (CB), Cd for cadmium red (CR) and cadmium yellow (CY), Cu and Ti for permanent blue light (PBL), Cu for permanent green light (PBC and PGL), CN and C_2_ for primary red magenta (PRM), Ti, CN and C_2_ for primary yellow (PI), Ti for titanium white (TW), and Zn for zinc white (ZW).

### 2.2. Thickness Measurements with OCT

**Figure 2:**
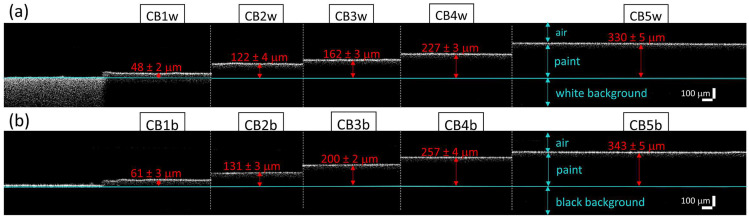
Assemblies of OCT tomograms acquired in Cobalt Blue (CB) paint laid on white (**a**) and black (**b**) backgrounds. For each area (1–5), thickness values are reported in red and calculated as the geometrical distance between the air–paint and the paint–background interfaces, with the latter highlighted by the light-blue line.

**Figure 3:**
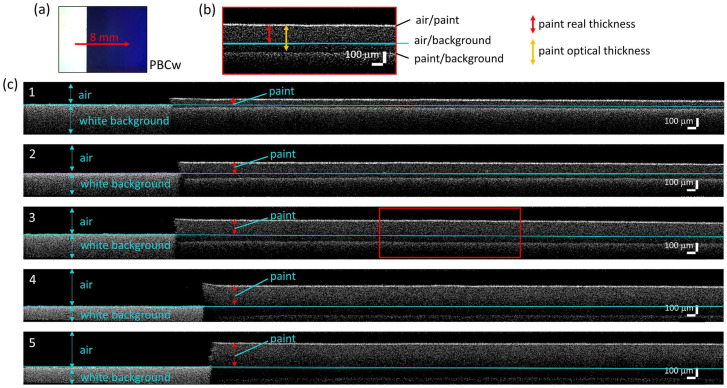
OCT results on Primary Blue Cyan paint laid on white background (PBCw); (**a**) microscope image of one of the five paint surfaces, with the red arrow indicating the location and length of the acquired section; (**b**) zoom-in of layer 3 delimited by the red rectangle in respective tomogram, enabling the assessment of the optical and real thicknesses used for calculating the refractive index of the acrylic paint; (**c**) OCT tomograms acquired on the five areas with increasing thickness (1–5), showing the transparency of the paint layer to the radiation probe.

Four xz tomograms (8 × 0.6 mm^2^, pixel size 3.5 μm^2^) were acquired in each painted area. Given the low transparency of most of the analysed paints, the layer thickness was measured by taking the signal generated at the interface air-background (visible at the edges of each painted area) as a reference, as shown in Figure 2. The thickness of each pictorial layer was calculated as the average over 20 values, resulting in a range between a minimum of 45 μm (area 1) to a maximum of 350 μm (area 5) for all acrylics. For each thickness, the error, i.e., the standard deviation, resulted below 6 μm for all areas, demonstrating the micrometric homogeneity of the paint layers. Only three acrylics, namely PBC (Figure 3), PRM, and PY, showed sufficient transparency to enable the evaluation of their refractive index, which was calculated by dividing the thickness measured with OCT by the real one. The resulting *n* values at 1300 nm, i.e., at the OCT radiation wavelength, are in the range of 1.35–1.40 for both PBC and PRM, and 1.45–1.50 for PY.

### 2.3. Thickness Measurements with Reflectance Spectroscopy

FORS and multi-spectral data were compared for each paint, as shown in Figure 3a–d. In the graphs, the average spectra of each painted area (1→5) are plotted together with the spectrum of the underlying substrate (white or black background). First and second derivatives were computed for all spectra to facilitate the identification of the spectral feature best representing the reflectance dependence on the material thickness. For all paints, the trend of the reflectance as a function of the thickness is expressed by an exponential function, following the equation:

(1) $$y=y_{0}+Ae^{R_{0}x}$$

where *y* = R%, *y_0_* = offset, *A* = initial value, *R*_0_ = growth constant, and *x* = layer thickness. 

**Figure 4:**
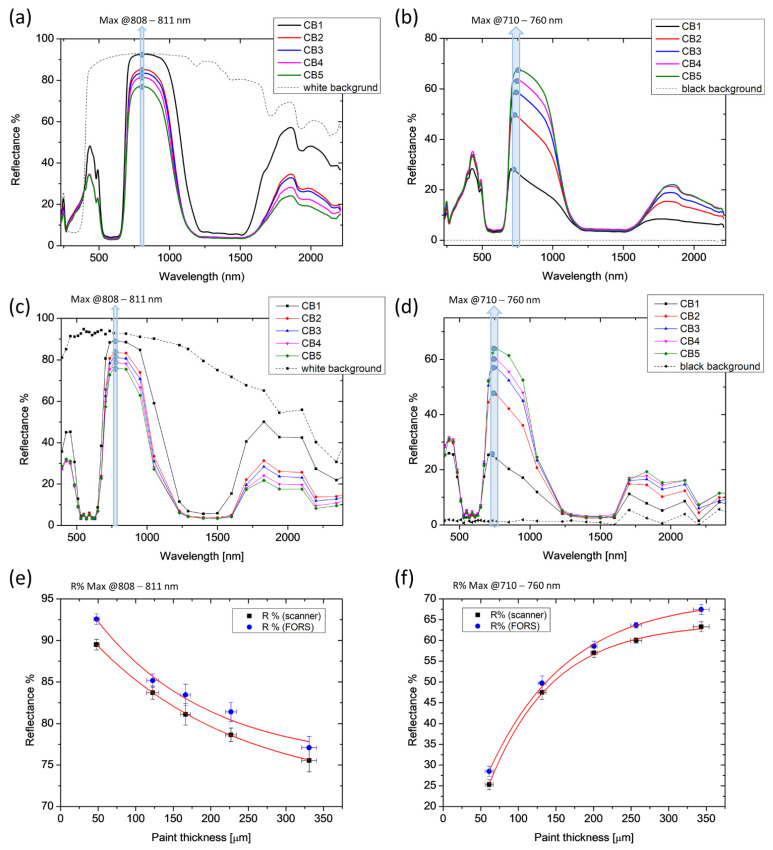
Results of diffuse reflectance spectroscopy on Cobalt Blue (CB) paint. Spectra acquired on each thickness layer (CB1-5) with FORS (**a**,**b**) and with the multi-spectral scanner (**c**,**d**) are reported with the spectra of the background (white or black). Maximum reflectance R% values in the 808–811 and 710–760 nm ranges are plotted as a function of the five OCT thicknesses (**e**,**f**). The length of the error bars is the standard deviation of each dataset. Red lines represent the fitting exponential functions.

In the case of CB paint, the maximum reflectance R% values in the 808–811 and 710–760 nm ranges were selected for the white and the black background series, respectively, and plotted as a function of the thickness (Figure 4e,f). We noticed that the presence of the black background affects the position of the point of maximum reflectance, causing a blue shift as the thickness of the paint layer decreases and becomes gradually more transparent. In the presence of the white background, however, the point of maximum R remains around 810 nm regardless of the thickness of the paint layer. The resulting exponential fit curves (coefficient of determination R^2^ > 0.98) show a good match between FORS and reflectance scanning results. 

**Figure 5:**
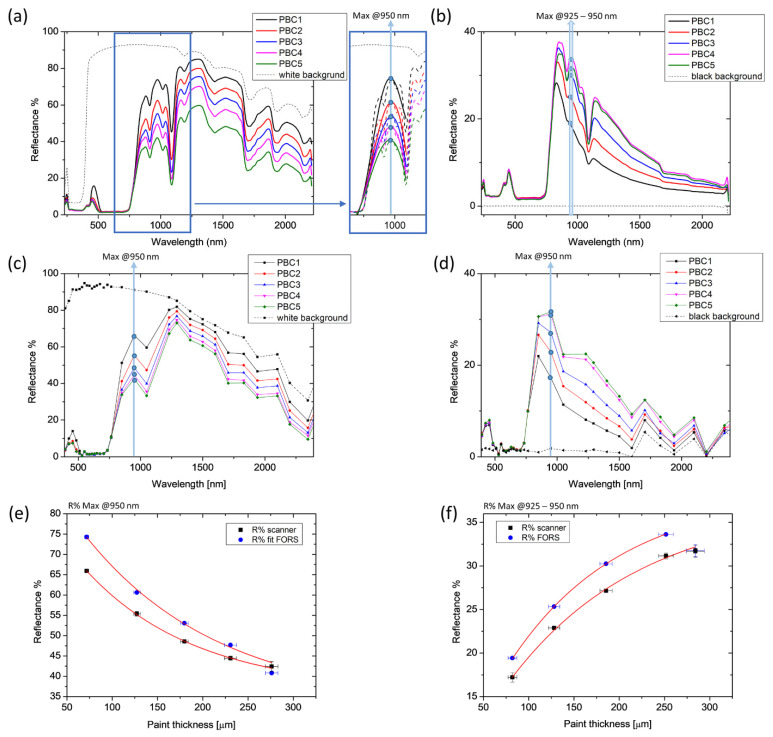
FORS (**a**,**b**) and multi-spectral scanner (**c**,**d**) results on Primary Blue Cyan (PBC) paint. The R% values at 950 nm are plotted with the five OCT thicknesses (**e**,**f**). The length of the error bars is the standard deviation of the dataset. Red lines represent the exponential fitting of the experimental points.

The spectral feature selected for the analysis in the FORS spectra was not always identifiable in the spectra from the spectral cube due to the significantly lower spectral resolution of the multi-spectral scanner. Therefore, in order to evaluate the applicability of the proposed method in multi-spectral imaging mode, matching key points were found in the two datasets. With this aim, the results of PBC laid on the white background are shown in Figure 5 as an example. The spectral region between 600 and 1200 nm was chosen as significant for our computation: the multi-peak FORS spectra were fitted with a 5th-degree polynomial (Figure 5a) to reconstruct the shape of the multi-spectral spectrum. Maximum reflectance at 950 nm was then considered for both datasets. The resulting exponential fitting functions of the two DRS data show good accordance (Figure 5e). The same maximum was considered for the paint laid on the black background (Figure 5b,d,f) without fitting the FORS spectra to retrieve the same spectral feature. In this case, the reflectance measured on the thickest layer (PBC5 = 284 ± 10 μm) was excluded from the exponential fitting calculation (Figure 5f), since it clearly deviated from the increasing trend, being lower than that of PBC4. This measurable thickness threshold has also been found in other pigments for thicknesses exceeding 270 microns. Remarkably, this limit of detectability was exclusively found in the FORS spectra. This is possibly due to the different measurement configurations between the two DRS modalities, which results in a greater homogeneity of illumination and, therefore, depth of detection achievable with the multi-spectral scanner than with fibre optics.

**Figure 6:**
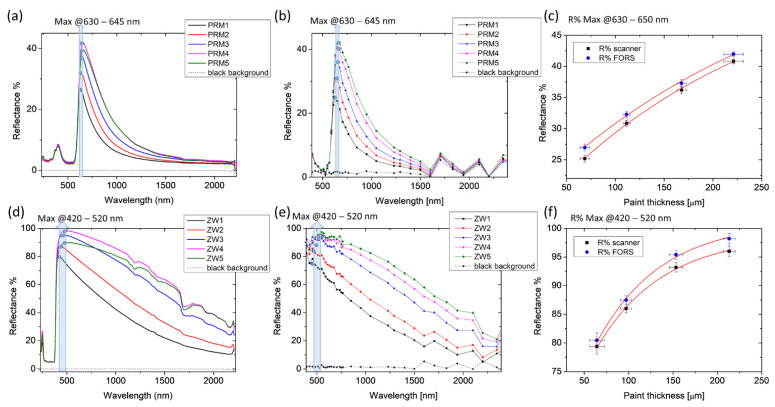
FORS (**a**,**d**) and multi-spectral scanner (**b**,**e**) results on Primary Red Magenta (PRM) and Zinc White (ZW) paints. R% values are plotted with the five OCT thicknesses (**c**,**f**). The length of the error bars is the standard deviation of the dataset. Red lines represent the exponential fitting of the experimental points.

Optimal agreement between FORS and multispectral data was found in acrylics showing high transparency in the NIR range. As an example, results on PRM on a black background are shown in Figure 6a–c. As for highly scattering pigments, such as ZW shown in Figure 6d–f, an exponential fit curve could be derived only in the presence of the black background. Additionally, in this case, the detection limit found with FORS is 250 microns for both PRM and ZW, corresponding to the thickness of area 5.

As expected, in the presence of the white background, the reflectance of the identified spectral feature was found to decrease exponentially with increasing paint thickness for all acrylics. In contrast, in the presence of the black background, the reflectance was found to increase exponentially. The fitting parameters of function (1) for the ten acrylic paints, together with the considered spectral feature, are summarized in Table 3. In few cases, the high scattering or absorption properties of some pigments made it impossible to identify the spectral feature of interest for the application of the exponential fit. Specifically, the proposed method could not be applied to CR, CY, PBL, TW and ZW laid on a white background and PGL laid on a black background. In all the other cases, the quality of the fitting (coefficient of determination R^2^) resulted in a value higher than 0.98.

**Figure 7:**
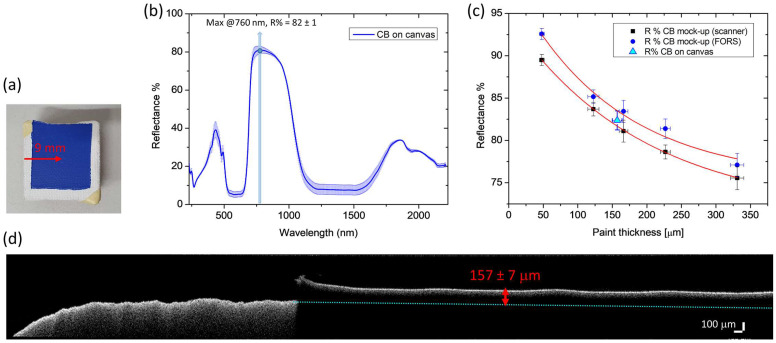
FORS and OCT results on CB laid on canvas: bright field image of the sample (**a**), with the arrow indicating the position and length of the OCT profile; averaged FORS spectrum (**b**) with a maximum at 760 nm considered for the analysis—the length of the error bars is the standard deviation; exponential functions previously obtained in the mock-up from FORS and scanner spectra, with R% measured on canvas sample reported in light blue (**c**); OCT tomogram of the paint layer with the measured thickness (**d**).

Finally, the proposed method was tested on a sample of cobalt blue laid on canvas (Figure 7a). In the FORS spectrum (Figure 7b), obtained from the average of nine spectra, the maximum at 760 nm, previously identified on the mock-up for CB, resulted in R% = 82 ± 1. The corresponding thickness obtained using the exponential functions derived from both FORS and scanner analysis ranges from 145 to 178 μm (Figure 7c), which includes the actual thickness range measured with OCT, i.e., 150–165 μm (Figure 7d).

## 3. Materials and Methods

### 3.1. The Painting Mock-Up

**Figure 8:**
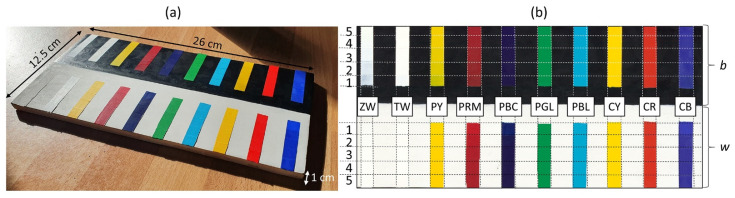
Acrylic painting mock-up: (**a**) photograph and **(b**) RGB image acquired with the multi-spectral scanner. For each acrylic, laid on the black (b) or white (w) backgrounds and labelled with the respective acronym (Table 1), the five areas with the increasing thickness (1→5)are delimited by the horizontal dotted lines.

The acrylic paints (extra-fine acrylic colours—Maimeri Brera^TM^, Milan, Italy) selected for this study are reported in Table 2, with their commercial code and chemical composition (as declared by the manufacturer). The surface of the wooden support, a tablet with size 12.5 × 26 × 1 cm^3^, was prepared with a layer of acrylic primer (Lefranc Bourgeois^TM^, Paris, France), which was accurately smoothed with fine-grit sandpaper. Half of the surface was covered with black acrylic paint (Carbon Black—PBk7—77266), as shown in Figure 8. Each acrylic colour was laid on five adjacent areas of 1 cm^2^ (1–5 in Figure 8b) with increasing thicknesses. To this aim, a nearly 50 μm thick tape (Kapton Cypress—DuPont^TM^, Wilmington, DE, USA) was used as a reference, i.e., depending on the desired thickness, 1 to 5 layers of tape were overlapped on both sides of the area to be painted. A glass spatula was used to spread the pigments in the various thicknesses in order to make their surface as even as possible.

The proposed method was finally tested on a sample made of a canvas industrially prepared with a gypsum-based primer and covered with a layer of Cobalt Blue acrylic paint.

### 3.2. Micro-Raman Spectroscopy

In order to enhance the detection of Raman signals emitted by high fluorescent paints such as Cadmium Yellow (CY), Cadmium Red (CR), and Primary Red Magenta (PRM), Raman analysis was carried out with two devices using different excitation wavelengths. 

The first device is a Renishaw InVia 0310–02 System based on a continuous Nd:YAG laser excitation source at 532 nm. The diameter of the laser spot on the sample was diffraction limited to 1 μm by the objective lens (50×). The system is equipped with a Leica microscope (DMI 3000 M) and an electrically cooled CCD camera. The laser power was set between 0.15 and 0.30 mW. 

The second device is a Renishaw Raman microscope RM1000 system coupled with an optical Leica DM LM microscope. The system is equipped with a refrigerated CCD camera and a CW He-Ne laser emitting at 632 nm, operating at a power of 3–30 mW, with a probing depth of 2 μm.

For all measurements, the spectral resolution was set at 4 cm^−1^ with an integration time in the range 10–60 s, the final spectra resulting from the accumulation of three individual ones.

### 3.3. Laser-Induced Breakdown Spectroscopy (LIBS)

The setup for LIBS analysis comprises a Q-switched Nd:YAG laser operating at its 4th harmonic at 266 nm, with a pulse duration of 15 ns and a repetition rate of 1 Hz. An f = 10 cm lens allows for fluences up to 6.6 J/cm^2^. The luminous emission was collected and dispersed by a 0.30 m spectrograph with a 1200 lines/mm grating (TMc300, Bentham, London, UK) coupled to an intensified charged coupled device (ICCD, 2151 Andor Technologies, Belfast, UK). Spectra were recorded at a 0.2 nm resolution, with a temporal gate of 3 μs and a delay of 500 ns, in order to avoid the continuous Bremsstrahlung emission. The laser beam was directed toward the surface of the samples at an angle of 45° by using different mirrors. A cut-off filter of 300 nm was placed at the entrance of the spectrograph to reduce the scattered laser light and avoid second-order diffraction. The shot-to-shot fluctuation of laser pulse energy was less than 10%. The spectra resulted from summing the emissions of the ablation products after five successive laser pulses, a number that provided a good signal-to-noise ratio.

### 3.4. Spectral-Domain Optical Coherence Tomography (Sd-OCT)

The OCT device used in this study is a Thorlabs Telesto-II, working in the 1300 nm regime, with an axial resolution of 5.5 μm in air and a lateral resolution of 13 μm. The maximum field of view (FOV) is 10 × 10 mm^2^, with a 3.5 mm imaging depth. The system is controlled via a 64-bit software running on a high-performance computer. The 3D scanning probe with an integrated video camera allows for high-speed imaging (76 kHz) for rapid volume acquisition and live display. The sample stage provides XY translation and rotation of the sample along with the axial travel of the probe. The 2D tomograms were acquired with a pixel size of 3.5 μm^2^. 

### 3.5. Fibre Optics Reflectance Spectroscopy (FORS)

FORS spectra were acquired with a Zeiss Multi-Channel Spectrometer, including two modules, the MCS601 UV-NIR C and the MCS611 NIR 2,2, with spectral sensitivity in the 190–1015 nm and 900–2200 nm range, and a spectral resolution of 0.8 nm/pixel and 5 nm/pixel, respectively. The size of the illumination spot was ∅ = 3 mm, and the illumination/observation geometry was 45°/0°. The output signal was processed through dedicated software, providing also CIEL*a*b* coordinates with standard D65 illuminant and 2° observer. The calibration procedure was performed following CIE indications for non-contact spectrophotometric measurements by measuring a certified white 100% reflectance reference standard (Spectralon, Labsphere^TM^, North Sutton, PA, USA) and background noise. 

For this specific application, it was essential to obtain spectra with reproducible reflectance, as the absolute intensity value is the core physical quantity to prove our thesis. Therefore, we defined an optimized procedure to both stabilize the signal intensity at each measurement and minimize fluctuations between the two modules. For each area (each of the five thicknesses), five reflectance spectra were measured in five different points with three consecutive acquisitions interleaved with dark correction by automatic closure of the device shutter. 

For each area, five averaged spectra (over three acquisitions to check the signal stability) were obtained, which were then averaged to obtain a single spectrum for each thickness, together with the standard deviation. The reflectance of the white and black backgrounds was also measured following the same procedure. Therefore, a total of 1530 spectra were acquired on the painting mock-up (510 spectra for each measured point). Spectral data were processed in OriginLab environment.

### 3.6. Reflectance Imaging Spectroscopy (RIS)

The multispectral scanner [36] developed at CNR-INO operates in the range 395–2550 nm providing simultaneously 32 narrow-band images (16 VIS + 16 NIR) and pointwise spectral information. The simultaneous movement of both the lighting system and collecting optics, placed in a 45°/0° illumination/detection geometry, allows for uniform illumination and minimal heating of the surface. During the scanning, an autofocus system ensures the optimal target-lens distance. The system has a 250 μm sampling step (4 points/mm) and 500 mm/s maximum speed. A proper calibration procedure was performed by measuring a certified white 100% reflectance reference standard (Spectralon™) and background noise, following CIE indications for non-contact spectrophotometric measurements. The spectra reported in this work were extracted from the spectral cube using an in-house developed software. Each spectrum is averaged over an area of ∅ = 8 mm selected on each painted region. Given that the system has a 250 μm sampling step (4 points/mm), each spectrum is averaged over ~805 pixels.

## 4. Conclusions

The chemical characterization of the considered ten acrylic paint materials was obtained by LIBS and micro-Raman spectroscopies, providing complementary information for the identification of elemental and molecular compounds in each paint and highlighting, in some cases, discrepancies with the chemical composition declared by the manufacturer. 

The results obtained with the proposed method show that there is a clear correlation between the spectral response of the diffuse reflectance and the micrometric thickness of acrylic paint layers. Using two DRS modes (FORS and multispectral scanner), it was possible to identify significant spectral features (maxima and inflexion points) where the exponential decay of the reflectance on the layer thickness was evident. The resulting exponential fitting functions showed good agreement between the two techniques, both showing opposite trends depending on the substrate (white or black) for all paints. The fitting function defined for cobalt blue was used to estimate the thickness of a layer of paint spread on canvas, which was consistent with the thickness measured by OCT.

Measuring the thickness of a pictorial layer from its spectral characteristics can overcome some limitations of the OCT technique. Knowing a priori the refractive index of the material in order to correct optical distances in OCT tomograms is often not possible, especially for pigment mixtures typically found on paintings. Here, we have shown that the proposed method allows the measuring of the thickness of ten acrylic paints, including optically opaque pigments that cannot be measured by OCT, such as CY and CR (only in the presence of a black background), without the need to know their refractive index.

In this work, the feasibility of the method was demonstrated, assuming a single-layer homogeneous paint was present. For more complex situations, such as layers stratifications or pigment mixtures in painted artworks, more advanced techniques for feature extraction can be used [37]. Moreover, a systematic study based on specific spectral reflectance models simulating the painting composition and structure should be developed, considering the optical behaviour of each component. The presence of multilayers or pictorial mixtures, commonly found in paintings, requires the creation of a much larger database. In this view, approaches based on AI could be tested. An interesting development of the technique is the possibility of obtaining thickness maps on painted surfaces using hyperspectral reflectography, which offers spectral resolution comparable to FORS, but over large scanning areas.

## Figures and Tables

**Table 1:** Summary of elemental and molecular composition of the acrylic paints as found by LIBS and micro-Raman spectroscopies. The main elemental components and the Raman characteristic bands are indicated in bold.

Paint	Identified Elemental Components by LIBS	Identified Raman Bands [cm^−1^] and Relative Intensities *. In Brackets the Excitation Wavelength
**CB**	Mg, Si, **Co**, **Al**, **CN**, Ca, Sr, C_2_, Na	**198 m, 408 w, 512 m, 609 w, 750 w,** 1007 m, 1150–1200 w, 2411 s, 2800–3100 s **(λ_exc_ = 532 nm)**
**CR**	Mg, Si, **Cd**, Al, **CN**, Ca, Sr, CH, **C_2_**, **Ba**, Na	**136 s, 200 s, 269 s, 488 w, 587 s,** 841 w, 985 w, 1007 w, 1150–1200 w, 1305 w, 1452 m **(λ_exc_ = 632 nm)**
**CY**	Mg, **Si**, **Cd**, Al, **CN**, Ca, Sr, CH, **C_2_**, **Ba**, **Na**	**212 s, 309 s, 353 w,** 600 s, 841 w, 985 w, 1007 s, 1150–1200 w, 1305 m, 1449 s, 1728 w **(λ_exc_ = 632 nm)**
**PBC**	Mg, Si, Al, **Cu**, **CN**, Ca, Ti, CH, **C_2_**, **Na**	**231 w, 255 w,** 482 w, **590 m, 680 m, 747 w,** 837 w, 841 w, **951 w**, 1007 w, **1037 w**, 1106 w, **1143 w**, 1150–1200 w, 1305 m, **1341 w, 1451 m, 1527 s, 1595 w, 2672 w, 2870 w, 2976 w, 3056 w (λ_exc_ = 532 nm)**
**PBL**	Mg, Si, Al, **Cu**, CN, Ca, **Ti**, Sr, C_2_, Na	**142 w, 231 m, 255 m, 433 s,** 482 w, **590 s, 609 s, 680 s, 747 w, 831 w**, 841, **951 w**, 1007 w, **1037 w, 1143 m**, 1150–1200 w, **1200 w, 1341 s, 1451 s, 1527 s, 1595 w, 2870 w, 3056 w (λ_exc_ = 532 nm)**
**PGL**	Mg, Si, **Cd**, Al, **Cu**, CN, Ca, Sr, CH, C_2_, **Ba**, Na	**162 w, 505 w**, 620 w, **685 s, 818 m, 978 w**, 985 w, 1007 w, **1080 m**, 1150–1200 m, **1200 m, 1284 s, 1340 m, 1388 s, 1503 s, 1536 s (λ_exc_ = 532 nm)**
**PRM**	Mg, Si, Al, **CN**, Ca, CH, **C_2_**, **Na**	841 m, 1007 w, 1150–1200, 1240 m, **1316 s, 1570 s, 1592 s, 1645 s (λ_exc_ = 632 nm)**
**PI**	Mg, Si, Al, **Ti**, **CN**, **Ca**, C_2_, Na	**186 m, 223 w, 261 w, 318 w, 360 w, 401 w, 525 w**, 600 w, **623 w, 646 w, 802 m, 922 w, 1066 w**, 1090 m, **1162 m**, 1150–1200 m, **1171 m, 1266 s, 1326 s, 1351 s, 1402 s, 1489 s, 1513 s, 1500 m,** 1593 s, **1667 w (λ_exc_ = 532 nm)**
**TW**	Mg, Si, **Ti**, CN, Ca, C_2_, Na	**138 m, 230 m, 445 s, 609 s,** 841 w, 1007 w, 1452 w, 2900–3100 s **(λ_exc_ = 532 nm)**
**ZW**	Mg, **Zn**, **CN**, Ca, CH, **C_2_**, **Na**	**330 w, 381 w, 435 s**, 620 m, 841 m, 1007 s, **1075 w, 1150 m,** 1150–1200 m, 1449 m, 1452 s, 1728 m, 2800–3100 s **(λ_exc_ = 532 nm)**

* s: strong; m: medium; w: weak.

**Table 2:** List of the ten analysed acrylic paints with their chemical composition and commercial code.

Paint with Acronym	Chemical Composition and Commercial Code (Maimeri Brera™)
Cobalt Blue (CB)	Cobalt(II) Aluminate [CoAl_2_O_4_], PB28—77346
Cadmium Red Medium (CR)	Cadmium Selenide Sulphide [Cd_2_SSe], PR108—77202
Cadmium Yellow Medium (CY)	Cadmium Sulphide [CdS], PY35—77205
Primary Blue Cyan (PBC)	Copper Phthalocyanine β [C_32_H_16_CuN_8_], PB15:3—74160
Permanent Blue Light (PBL)	Titanium Dioxide [TiO_2_] PW6—77891, Chlorinated Phthalocyanine [C_32_HCl_15_CuN], PG7—74260, Copper Phthalocyanine β [C_32_H_16_CuN_8_], PB15:3—74160
Permanent Green Light (PGL)	Arylide yellow, PY97—11767, Titanium Dioxide [TiO_2_], PW6—77891, Chlorinated Phthalocyanine [C_32_HCl_15_CuN], PG7—74260
Primary Red Magenta (PRM)	Quinacridone [C_20_H_12_N_2_O_2_], PV19—73900
Primary Yellow (PI)	Arylide Yellow, PY97—11767
Titanium White (TW)	Titanium Dioxide [TiO_2_], PW6—77891
Zinc White (ZW)	Zinc Oxide [ZnO], PW4—77947

**Table 3:** Fit parameters of the exponential function (1) describing for each paint the dependence of the reflectance of the spectral feature identified with FORS and multi-spectral scanning on the layer thickness.

Paint	Background	Spectral Feature and Range [nm]	Technique	Fitting Parameters			Adjusted R^2^
				$y_{0}$	A	$R_{0}$	
**CB**	w	max @808–811	FORS	75.29	23.78	−7 × $10^{-3}$	0.98
			Scanner	71.46	23.13	−5 × $10^{-3}$	
	b	max @710–760	FORS	70.1	−74.28	−9 × $10^{-3}$	0.99
			Scanner	64.16	−79.75	−11 × $10^{-3}$	
**CR**	w	n.a.	FORS	n.a.	n.a.	n.a.	n.a.
			Scanner	n.a.	n.a.	n.a.	
	b	flex @1400	FORS	94.19	−36.15	−9 × $10^{-3}$	0.98
			Scanner	87.7	−36.6	−10 × $10^{-3}$	
**CY**	w	n.a.	FORS	n.a.	n.a.	n.a.	n.a.
			Scanner	n.a.	n.a.	n.a.	
	b	flex @1705	FORS	65.93	154.23	−25 × $10^{-3}$	0.99
			Scanner	69.01	−97.52	−19 × $10^{-3}$	
**PBC**	w	max @950	FORS	33.03	66.86	−6 × $10^{-3}$	0.99
			Scanner	36.52	53.01	−8 × $10^{-3}$	
	b	max @925–950	FORS	38.46	−37.28	−8 × $10^{-3}$	0.99
			Scanner	37.21	−34.98	−7 × $10^{-3}$	
**PBL**	w	n.a.	FORS	n.a.	n.a.	n.a.	n.a.
			Scanner	n.a.	n.a.	n.a.	
	b	max @1230	FORS	91.73	−127.65	−24 × $10^{-3}$	0.99
			Scanner	86.42	−78.84	−18 × $10^{-3}$	
**PGL**	w	max @1050	FORS	69.17	22.57	−8 × $10^{-3}$	0.99
			Scanner	64.72	22.90	−8 × $10^{-3}$	
	b	n.a.	FORS	n.a.	n.a.	n.a.	n.a.
			Scanner	n.a.	n.a.	n.a.	
**PRM**	w	flex @610	FORS	45.97	−7.06	−4 × $10^{-3}$	0.97
			Scanner	43.76	−3.72	−5 × $10^{-3}$	
	b	flex @610	FORS	29.93	−20.94	−6 × $10^{-3}$	0.99
			Scanner	29.34	−23.34	−10 × $10^{-3}$	
**PY**	w	max @2010	FORS	32.97	29.38	−6 × $10^{-3}$	0.99
			Scanner	33.13	24.45	−6 × $10^{-3}$	
	b	max @550–570	FORS	91.12	−45.4	−6 × $10^{-3}$	0.99
			Scanner	80.9	−42.05	−9 × $10^{-3}$	
**TW**	w	n.a.	FORS	n.a.	n.a.	n.a.	n.a.
			Scanner	n.a.	n.a.	n.a.	
	b	max @1230	FORS	100.86	−72.65	−17 × $10^{-3}$	0.99
			Scanner	89.51	−60.77	−19 × $10^{-3}$	
**ZW**	w	n.a.	FORS	n.a.	n.a.	n.a.	n.a.
			Scanner	n.a.	n.a.	n.a.	
	b	max @420–520	FORS	101.20	−50.74	−14 × $10^{-3}$	0.99
			Scanner	98.77	−46.81	−14 × $10^{-3}$	

## Data Availability

The data presented in this study are available on reasonable request from the corresponding author.

## Supplementary Materials

The following supporting information can be downloaded at: https://www.mdpi.com/article/10.3390/molecules28124683/s1, Figure S1: Chemical characterization of the acrylic paints with LIBS and micro-Raman spectroscopy.

## Sample Availability

Samples of the compounds are available from the authors under reasonable request.
