# Supplementary material for: Reflectance Spectroscopy as a Novel Tool for Thickness Measurements of Paint Layers
Source: Molecules. 2023 Jun 9;28(12):4683. doi: 10.3390/molecules28124683 (PMC10301771; doi:10.3390/molecules28124683)
Supplement: Supplementary file 1 [file molecules-28-04683-s001.zip › molecules-2409663-supplementary.pdf]

Reflectance spectroscopy as a novel tool for thickness measurements of paint layers

A. Dal Fovo[1], M. Martínez-Weinbaum[2], M. Oujja[2], M. Castillejo[2], R. Fontana[1]

[1] Consiglio Nazionale delle Ricerche-Istituto Nazionale di Ottica (CNR-INO), Largo E. Fermi 6, 50125 Florence, Italy

[2] Instituto de Química Física Rocasolano, Spanish National Research Council (CSIC), C/ Serrano 119, 28006 Madrid, Spain

Chemical characterization of the acrylic paints with LIBS and micro-Raman spectroscopy

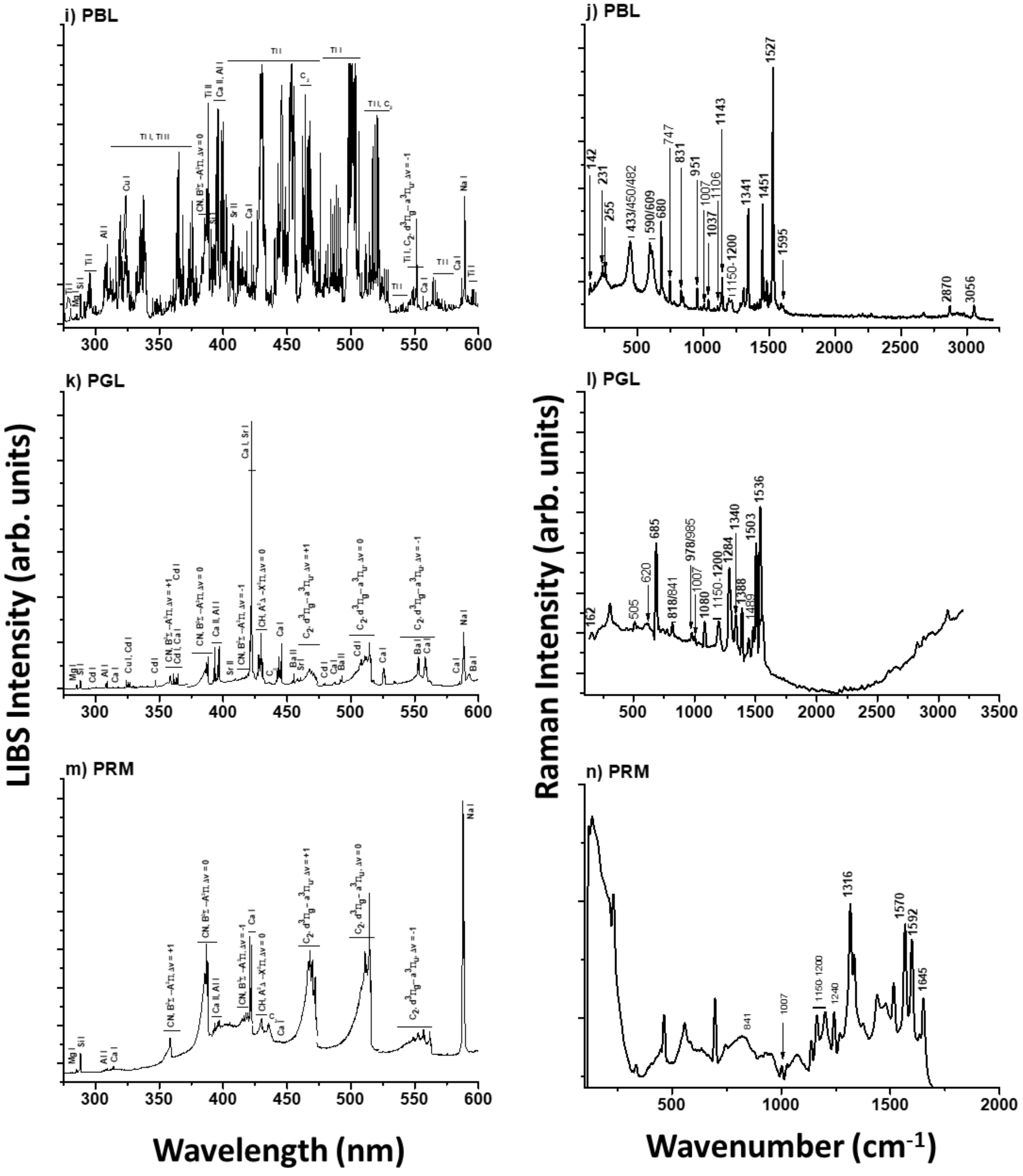

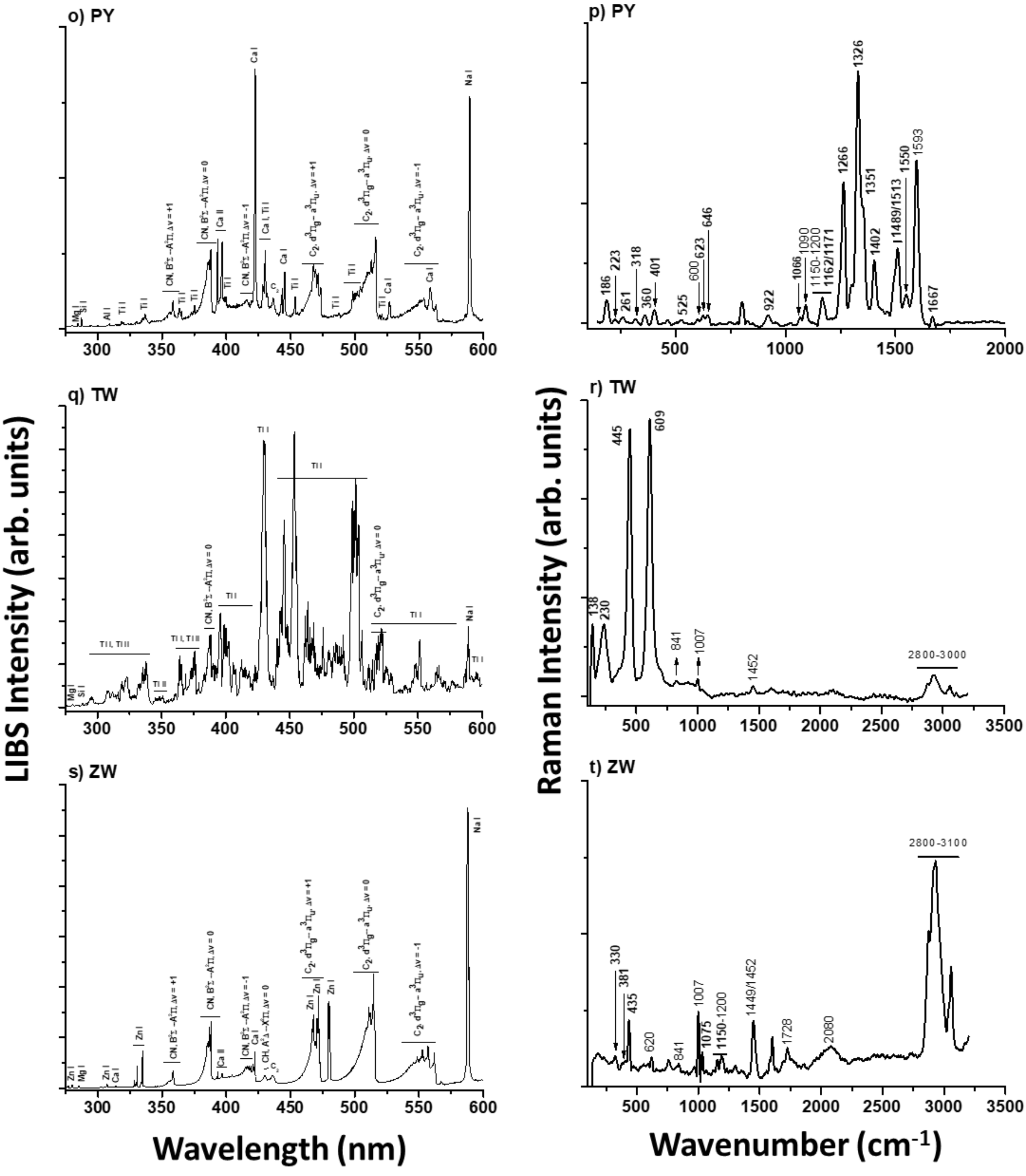

Figure S1 (i-t). LIBS (left column) and micro-Raman (right column) spectra of permanent blue light (PBL) (i,j), permanent green light (PGL) (k,l), primary red magenta (PRM) (m,n), primary yellow (PI) (o,p), titanium white (TW) (q,r) and zinc white (ZW) (s,t) acrylic paintings. The micro-Raman spectra were baseline subtracted.
